# Titanium: An Unusual Allergen With Various Presentations—A Retrospective Cohort Study

**DOI:** 10.1111/cod.70021

**Published:** 2025-08-26

**Authors:** Danny Daniely, Valentina Zemser‐Werner, Roy Gilon‐Omer, Jonathan Bar, Dan Slodownik

**Affiliations:** ^1^ Department of Dermatology Tel Aviv Sourasky Medical Center Tel Aviv Israel; ^2^ School of Medicine Tel Aviv University Tel Aviv Israel

## Abstract

**Background:**

Titanium, a rare allergen tested within a designated metal series, has a unique exposure profile, warranting evaluation in specific clinical scenarios. Our study aims to characterise the clinical features of patients with Titanium sensitisation.

**Methods:**

A retrospective cohort analysis was conducted on 255 patients patch tested with the metal series at a tertiary contact dermatitis clinic between 2012 and 2024. Clinical characteristics and sensitisation patterns were evaluated.

**Results:**

The metal series was performed in several main indications: occupational scenarios, exposure to jewellery, post‐dental or orthopaedic implants and tattoo‐related dermatitis. The cohort predominantly comprised Caucasian females (57%) with a mean age of 51. Only ~20% of cases with occupational exposure and post‐orthopaedic implant elicited a positive reaction to metals. 67% of patients in the latter group and all patients with tattoo‐induced dermatitis would have been missed unless the metal series was performed. A positive relevant reaction to titanium oxalate was evoked in 5% of cases; 38% were associated with jewellery. Co‐sensitisation with other metals including nickel, cobalt and vanadium was common. Titanium typically caused dermatitis adjacent to the contact site, mainly involving fingers and hands. Two cases associated with medical implants developed a generalised rash, 1 of whom was diagnosed with Bullous pemphigoid (BP), confirmed by immunofluorescence.

**Conclusions:**

While nickel, cobalt and chrome are considered common allergens, we hypothesise that sensitisation to other metals, including titanium, will become more prevalent in the following years through exposure via medical devices and implants as well as recreationally via ‘nickel free’ jewellery and tattoos. Titanium‐induced BP is an intriguing phenomenon that should be further investigated, on the verge between allergology and immunology.

## Graphical abstract

Titanium, a rare allergen, should be tested in the suitable clinical setting, taking into consideration the anticipated rise in exposure through medical equipment, implants and recreational ‘Nickel free’ jewellery and tattoos. Titanium induced BP is an intriguing phenomenon that should be further explored, on the verge between allergology and immunology.

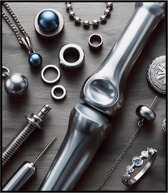

## 1 Introduction

First discovered in 1791, titanium (Ti) is a light yet relatively strong metal, showing a high strength/density ratio [1]. When exposed to air, it forms a titanium dioxide TiO_2_ 1–2 nm thick coating within less than a millisecond. TiO_2_ acts as a barrier between the metal and air, contributing to its corrosion‐resistant properties [2]. However, mechanical damage to the TiO_2_ film exposes the metal to oxidants and water, facilitates corrosion and hydrogen embrittlement, reducing the stress intensity threshold for crack propagation [3].

Titanium has several routes of exposure. TiO_2_ is a white powder and is the most ubiquitous form in medicine. In the nanoscale form, it has a dual behaviour towards light [4], absorbing and scattering photons, commonly used as a physical ultraviolet and visible light absorber in sunscreens [5, 6], as well as in cosmetics and makeup for its high opacity and brightness.

Titanium is considered biocompatible, wear resistant, non‐corrosive and integrates well in the bone tissue [7]. It is commonly used in the medical field in the formation of dental and orthopaedic implants, cardiac pacemakers, cardiac stents and neuromodulatory devices [8, 9]. Additionally, titanium is used in the formation of ‘Hypo‐allergic’ jewellery, mainly rings and body piercings for patients with nickel allergy, along with aluminium, vanadium and gold [10].

From an occupational point of view, titanium is utilised to produce strong lightweight alloys in the aerospace, military and automotive industry as well as in desalination plants and in the agri‐food industry.

Ti allergy is considered extremely rare, primarily since its main form TiO_2_ does not penetrate the skin [11], nor is it absorbed via the gastrointestinal tract. In addition, like other transition metals, it does not form covalent protein modifications accessible to the immune system. Nonetheless, as clinical cases of suspected Ti allergy emerged, it has been demonstrated that Ti ions and Ti salts from corrosion products can activate macrophages and elicit an immune response [12, 13].

Diagnosing Ti allergy remains a controversial issue. While patch testing was originally conducted with the inert form TiO_2_, hardly eliciting any reactions, in recent years several Ti salts, including Ti oxalate hydrate, Ti lactate, Ti isopropoxide and Ti citrate, appear to be superior candidates for patch testing in suspected Ti allergy cases [14].

Given the expanding use of titanium and the evolving understanding of its allergenic potential, our study aims to characterise the exposure patterns and clinical characteristics of patients with titanium contact allergy.

## 2 Methods

### 2.1 Participants

This retrospective study includes 255 patients of any age who were referred to a designated patch test clinic in Tel Aviv Sourasky Medical Centre dermatology department between 1.2012 and 3.2024 and were patch tested with the metal series (Chemotechnique Diagnostics, Vellinge, Sweden, Data S1), with additional series as clinically indicated.

### 2.2 Patch Testing Protocol

Allergens were placed in IQ Ultra patch test units and immediately applied to the patients' upper back. Allergens were left in place for 2 days, and readings were performed on day (D) 4 for all patients. D7 readings were performed in 4 cases, only if the patient noticed a reaction and voluntarily arrived at the clinic. Positive reactions were scored as weak (+), strong (++) or extreme (+++) according to the International Contact Dermatitis Research Group and the European Society of Contact Dermatitis criteria [15]. Clinical relevance was determined based on the European Society of Contact Dermatitis criteria [15]. Titanium was patch tested using titanium nitride 5.0% pet and titanium oxalate hydrate 5.0% pet.

### 2.3 In Vitro Testing

One patient underwent memory lymphocyte immuno‐stimulation assay (MELISA) as an adjunct diagnostic tool (Data S1).

### 2.4 Exclusion Criteria

The exclusion criteria include patients who did not complete the patch test, patients who were being treated with systemic corticosteroids or another immunomodulating agent (including azathioprine, cyclosporine, mycophenolate mofetil) during the last month, or received phototherapy less than 1 month prior to patch testing.

### 2.5 Statistical Analysis

Categorical variables were compared using Fisher's exact test. Two‐sided *p*‐values < 0.05 were considered statistically significant. R 4.4.2 and RStudio 2024.12.1 were used for the statistical analysis.

## 3 Results

Two hundred and fifty‐five out of 6550 referred patients (3.9%) were patch tested with the metal series. Two hundred and thirty‐six patients were tested with the European baseline series (EBS) as well (92%). Demographics are summarised in Table 1.

**TABLE 1:** Demographic and clinical characteristics (MOAHLFA* index in *Italics*) of patients tested with the metal series.

Characteristic	*N* (%)
*Male*	*110 (43%)* ** *OR 1.41*
*Occupational dermatitis*	*79 (31%)* ** *OR 6.7*
*Age over 40*	*173 (68%)* ** *OR 1.79*
12–18 years old	9 (3.5%)
Atopic triad	
*Atopic dermatitis*	*21 (8%)* ** *OR 0.44*
Asthma	51 (20%)
Allergic rhinitis	72 (28%)
Main location	
*Face (including mouth lips and eyelids)*	*84 (33%)* ** *OR 1.58*
*Hand*	*58 (23%)*
*Leg*	*30 (12%)*
Generalised	27 (11%)
Trunk	24 (9%)
Anogenital	2 (< 1%)** OR 0.1
Flexural	5 (2%)
Arm	16 (6%)
Neck	9 (4%)
Mean duration of symptoms (months)	34.4

* Abbreviations: A, % patients with atopic dermatitis; A^(2)^, % patients age 40 and above; F, % patients with face dermatitis; H, % patients with hand dermatitis; L, % patients with leg dermatitis; M, % male patients; O, % patients with occupational dermatitis.

** OR‐odds ratio statistically significant (*p* < 0.05) in comparison to the general patch tested population excluding patients tested with the metal series.

The cohort had a mean age of 51.1 years (±18.6), with a predominance of Caucasian females (57%). Compared to the general patch‐tested population, these patients were significantly more likely to be male (OR 1.41, *p* < 0.05), aged over 40 (OR 1.79), and tested due to occupational exposure (OR 6.7) or facial involvement (OR 1.58). They were less likely to have atopic dermatitis (OR 0.44) or anogenital dermatitis (OR 0.1) (Data S1).

The metal series was performed in several major indications:

1. Occupational exposure, including technicians, engineers, mechanics and jewellers—79 patients (31%). Sixteen had a positive metal reaction (20%).
2. A clinical skin reaction to jewellery—72 patients (28%), including 12 patients who were referred by a dentist prior to reconstructive treatments, and nine patients by an orthopaedic surgeon prior to a joint replacement. Forty three had a positive metal reaction (60%).
3. A clinical suspicion for a metal allergy causing a dental implant failure or a rash involving the oral mucosa adjacent to a dental implant—45 patients (17%). Ninteen had a positive metal reaction (42%).
4. Clinical suspicion for a metal allergy causing implanted joint failure or a localised/generalised rash post joint replacement—31 patients (12%). Six had a positive metal reaction (19%).
5. A rash involving tattoo—17 patients (7%). Five had a positive metal reaction (29%).
6. Other indications—11 patients (5%)—including rash adjacent to a cardiac pacemaker—4 patients, a rash adjacent to a belt knuckle—2 patients, a rash suspected to be caused by a shaving razor, wheelchair, sunscreen, hijab metal knuckle or post breast implant—1 patient each.

One hundred and twenty‐nine patients (51%) had no reaction at all. Among patients with a positive reaction, the mean number of reactions is 3.1. We assessed the rate of positive relevant reactions (PRR) to metals, whether among the EBS‐nickel sulfate 5.0% pet, potassium dichromate 0.5% pet, cobalt chloride 1.0% pet or among the metal series according to the clinical indication (Table 2).

**TABLE 2:** Positive relevant reactions to metals according to the clinical indication.

Indication	Number of PRR^a^	Number of PRR^a^ to nickel, cobalt or chrome	Additional number of reactions in the metal series
Occupational	36	12	24
Jewellery	104	43	61
Dental	48	18	30
Orthopaedic	16	3	13
Tattoo	6	0	6

^a^ PRR, positive relevant reaction.

Titanium oxalate hydrate elicited positive reactions in 13 patients (5%). All reactions occurred with titanium oxalate, none with titanium nitride. Clinical characteristics of patients with Ti sensitization are demonstrated in Table 3. Affected individuals were predominantly adult Caucasian females, with 38% linked to jewellery exposure. Co‐sensitization with other metals—particularly nickel, cobalt, and vanadium—was present in 77% of these cases.

**TABLE 3:** Clinical characteristics of patients with titanium sensitisation.

Indication	Gender	Age	Atopic dermatitis	Allergic rhinitis	Asthma	Duration of symptoms (months)	Location	Co‐sensitization
Jewellery	M	70	+	—	—	6	Finger	Manganese, cobalt chloride, ammonium persulfate
	F	53	—	—	+	1	Finger	Nickel, cobalt, vanadium
	F	22	—	—	—	3	Neck	Antimony oxide
	F	30	—	+	+	8	Finger	—
	M	71	—	—	—	7	Waist	Vanadium, manganese
Occupational	F	44	+	—	—	12	Hands	Nickel, tin, zinc, sodium thiosulfate aurate, thiuram mix
	M	54	—	—	—	18	Hands	Ferrous chloride
Post orthopaedic implant	F	68	—	—	—	8	Arms	Gold, vanadium, beryllium
	M	76	—	—	—	2	Generalised	—
Post dental implant	M	82	—	—	—	60	Generalised	—
	M	72	—	—	—	24	Mouth	Nickel, potassium dicyanoaurate, silver, vanadium, iridium
Sunscreen	F	74	—	—	+	12	Generalised	Potassium dichromate, nickel, palladium chloride, copper sulfate, iridium
Tattoo	F	29	—	—	+	3	Back	Nickel, cobalt chloride, ferric chloride, vanadium

Clinical presentations were primarily localised eczematous eruption in the contact site, mainly the fingers, hands or trunk. Interestingly, two patients developed a generalised rash following implant placement, one dental and one orthopaedic. One of those patients developed a generalised bullous eruption 1‐week post titanium‐based implant hip replacement. A +++ reaction to Ti oxalate was evoked on D4. Direct immunofluorescence conformed the diagnosis of bullous pemphigoid. MELISA test (Memory Lymphocyte Immunostimulation Assay) [16] was performed as well, showing a weakly positive reaction to titanium dioxide, as well as to zirconium sulfate, lead nitrate, silica dioxide and fine ground silica.

## 4 Discussion

The metal group is the most common group of allergens among the baseline series in Israel [17], and nickel sulfate is the most common allergen in North America and Europe [18, 19]. The designated metal series, composed of 30 metals, is rarely performed (~5% of cases in our cohort) although a previous study by our group demonstrated a relatively high added value, yielding a relatively low number needed to test for an additional reaction [20].

Patch testing is considered the gold standard for diagnosing metal induced ACD [21], with a preference for an additional delay reading on D7 [22]. However, it has several disadvantages, including a cytotoxic effect of metal salts on keratinocytes, thus confounding the results, causing irritant reactions, and allegedly serving as a potential sensitizer [23]. In addition, there is conflicting data regarding the most suitable metal salt composition for patch testing [24]. Thus, additional methods for diagnosis have emerged in the last decades, based on the activation and transformation of peripheral blood lymphocytes in vitro after incubation with metal ions [25]. While still considered controversial [26, 27] with a suggested high number of false positive reactions, they serve as an ancillary tool in diagnosing metal induced contact sensitisation.

In our cohort, the metal series was most commonly performed in an occupational scenario, yielding a relatively low added value, as most cases with metal induced occupational ACD were caused by common metals tested in the EBS. On the other hand, metals represented in the metal series were a common culprit in patients with jewellery provoked or post dental procedure ACD. It is worth noting that the majority of patients suffering from jewellery induced ACD are being tested with EBS alone, and only complicated recalcitrant cases are being tested with the metal series, hence eliciting additional reactions. We hypothesise that the increasing exposure to ‘nickel free’ jewellery [28] may shift metal sensitisation trends towards other metals including gold, silver, titanium, copper and platinum.

Tattoo‐induced sensitisation is an emerging and underrecognised source of metal exposure. Approximately 30% of Americans have at least one tattoo and 25% of permanent tattoo inks are composed mainly of inorganic metallic compounds, including iron oxide, titanium dioxide and barium sulfate [29, 30]. Additionally, organic inks, including phthalocyanines and xanthenes, form pigments by complexing with metals, mainly copper and molybdenum. Tattoo‐induced allergic reaction, caused by the pigment itself or its breakdown products [31], is a challenging diagnosis that should be further explored. In our cohort, all patients with suspected tattoo‐induced ACD to a metal component would have been missed unless the metal series was performed. However, since there are no governmental regulations over tattoo inks and ingredient reporting is not mandatory in Israel, these findings should be interpreted with caution and the diagnostic relevance of the patch test results remains inconclusive.

Metal sensitization by medical devices has been of increased interest in recent years, particularly by dental and orthopaedic implants, causing implant failure and skin disorders. There are several types of dental implants, most of them based on metal alloys: cobalt–chrome, stainless steel (nickel–chrome), noble metals (gold–palladium–platinum) or titanium–aluminium–vanadium (Ti_6_Al_4_V) [32]. In recent years, ceramic implants, composed mainly of zirconium dioxide, have been developed and are considered free of metals in the zero valent form. Orthopaedic implants are composed of either metallic biomaterials (stainless steel/titanium alloy (Ti_6_Al_4_V), cobalt–chrome–molybdenum), ceramic biomaterials (aluminium and zirconium oxide) or polymeric biomaterials (teflon, polyethylene or polimethylmetacrylate) [33]. Hoyos et al. [34] have recently described their experience with titanium patch testing. Surprisingly, none of their described cases of post‐implant Ti allergy were due to a dental implant, emphasising the importance of more studies in the field.

Among the metal series, titanium is a relatively rare allergen, positive in 5% of cases in our cohort, in concordance with a previous publication [14]. We examined all patients with titanium oxalate hydrate, which is considered the most reliable salt for patch testing [35]. Seventy seven percentage of cases were co‐sensitised with other metals, mostly nickel, cobalt and vanadium. Eleven out of 13 patients with a positive relevant reaction to titanium oxalate developed a local eczematous rash. Two patients developed a generalised rash, one of whom had eczematous features that might represent systemic contact dermatitis and one with bullous pemphigoid proven by immunofluorescence. Fuller et al. [36] have recently described a case of a generalised annular rash and keratoderma 10 weeks post Ti based hip implant surgery. Samuel et al. [37] have previously described a patient with BP induced by titanium containing knee implant, 2 months postoperatively, mainly distributed around the implanted knee. Our case is the first in the literature with a generalised distribution of BP, hence raising an intriguing association. In this regard, it should be noted that not only metal alloys might induce BP post joint replacement, as methacrylate in the bone cement was described to trigger BP postoperatively as well [38].

In addition to sensitization via dental and orthopaedic implants, titanium is considered to be the metal of choice in reconstructive surgery (screws, plates) and in cardiovascular devices [39, 40]. It was recently described to cause contact dermatitis adjacent to a cochlear implant [41]. Other modes of exposure include contact with eyeglasses frames [42], personal care products and via ingestion of foods (mainly sweets) [43].

Lastly, titanium dioxide, an inorganic UV filter in sunscreens, has not been described in the literature to elicit sensitisation via topical application [44]. One patient in our cohort with suspected sunscreen‐induced contact allergy reacted to the bioavailable titanium oxalate, a reaction considered of doubtful relevance.

In conclusion, we present our experience with titanium‐induced sensitisation.

Our study has several limitations. Day 7 readings were not routinely performed, potentially missing late‐reacting allergens such as titanium and allegedly misinterpreting an irritant reaction as allergic. Additionally, only one patient underwent MELISA testing, and titanium oxalate, the most reactive salt, was not included in the MELISA panel, limiting comparison.

Titanium, traditionally considered a biologically inert metal with minimal allergenic potential, was found to elicit positive, clinically relevant patch test reactions and should be tested in the suitable clinical scenario, especially considering the expected increase in exposure via medical devices [45] and nickel‐free jewellery.

## Conflicts of Interest

The authors declare no conflicts of interest.

## Supporting information


**Data S1:** cod70021‐sup‐0001‐Supinfo.docx.

## Data Availability

The data that support the findings of this study are available from the corresponding author upon reasonable request.
