# Supplementary material for: Titanium: An Unusual Allergen With Various Presentations—A Retrospective Cohort Study
Source: Contact Dermatitis. 2025 Aug 26;93(6):507–13. doi: 10.1111/cod.70021 (PMC12586302; doi:10.1111/cod.70021)
Supplement: Supplementary file 1 — Data S1: cod70021‐sup‐0001‐Supinfo.docx. [file COD-93-507-s001.docx]

**Supplements**

1. Metal series**,** Chemotechnique Diagnostics

| 1. | Aluminium(III)chloride hexahydrate | 2.0% pet |
| --- | --- | --- |
| 2. | Cobalt(II)chloride hexahydrate | 1.0% pet |
| 3. | Beryllium(II)sulfate tetrahydrate | 1.0% pet |
| 4. | Gold(I)sodium thiosulfate dihydrate | 2.0% pet |
| 5. | Copper(II)sulfate pentahydrate | 2.0% pet |
| 6. | Tin | 50.0% pet |
| 7. | Iridium(III)chloride trihydrate | 1.0% pet |
| 8. | Titanium(III)nitride | 5.0% pet |
| 9. | Mercury(II)amidochloride | 1.0% pet |
| 10. | ZINC CHLORIDE | 1.0% pet |
| 11. | Titanium(IV)oxalate hydrate | 5.0% pet |
| 12. | Sodium tetrachloropalladate(II) hydrate | 3.0% pet |
| 13. | Molybdenum | 5.0% pet |
| 14. | Vanadium(III)chloride | 1.0% pet |
| 15. | MANGANESE CHLORIDE | 2.0% pet |
| 16. | Zirconium(IV)chloride | 1.0% pet |
| 17. | Tungsten | 5.0% pet |
| 18. | FERRIC CHLORIDE | 2.0% pet |
| 19. | Ammonium hexachloroplatinate(IV) | 0.1% aq |
| 20. | Cadmium chloride | 1.0% aq |
| 21. | Indium(III)chloride | 10.0% aq |
| 22. | Nickel(II)sulfate hexahydrate | 5.0% pet |
| 23. | Palladium(II)chloride | 2.0% pet |
| 24. | Gallium(III)oxide | 1.0% pet |
| 25. | Ruthenium | 0.1% pet |
| 26. | SILVER NITRATE | 1.0% aq |
| 27. | ALUMINUM HYDROXIDE | 10.0% pet |
| 28. | Niobium(V)chloride | 0.2% pet |
| 29. | Tantalum | 1.0% pet |
| 30. | Potassium dichromate | 0.5% pet |

1. MELISA® (47)

Ten milliliters of peripheral venous blood were collected and centrifuged to provide the patients' autologous serum. Heat-inactivated autologous serum was used for the cultivation of lymphocytes. 30 ml of peripheral venous blood was collected and mixed with an equal amount of RPMI 1640 medium containing 10 mM HEPES, gentamycin, and glutamine. The blood was layered on a Ficoll-Paque gradient (Histopaque, Sigma Aldrich) and centrifuged at 600 *g* for 30 min. Mononuclear cells were collected from the interface, washed twice, then mixed with 5 ml of RPMI 1640 medium containing 20% of inactivated autologous serum. Plastic-adherent cells were partially depleted from leukocyte suspension by incubation on plastic surfaces for 40 min at 37 °C. After incubation, the lymphocytes were counted and diluted with RPMI 1640 enriched with 10% autologous serum and glutamine into a final dilution of 1 × 10 ^6^ cells/ml. Lymphocytes were cultivated for five days with metal salts solutions in an atmosphere of 5% CO _2_ in humidified air at 37 °C. All patients in this study were tested to nickel, chromium, iron, titanium (in the form of chloride and oxide), aluminum, molybdenum, copper, platinum, cobalt and zirconium. Details of the specific metal salts and their concentrations are described by Stejskal et al. Control cultures were incubated under the same conditions in the absence of metal salt solutions. As a positive control, lymphocytes were cultivated with Pokeweed mitogen (10 μg per ml, Sigma, USA). After five days' cultivation, lymphocyte cultures were split into two parts. One part was used to measure lymphocyte proliferation by  H thymidine incorporation (Perkin Elmer, USA), as described in the article by Stejskal et al. The second part was frozen at −20 °C to determine pro-inflammatory cytokine production.

1. Demographic and clinical characteristics (MOAHLFA* index in *Italics*) of patients who were patch tested with other series than the metal series (N=6295).

| Characteristic | N (%) |
| --- | --- |
| *Male* | *2202 (35%)* |
| *Occupational dermatitis* | *395 (6.3%)* |
| *Age over 40* | *3406 (54.1%)* |
| 12-18 years old | 207 (3.3%) |
| Atopic triad |  |
| *Atopic dermatitis* | *1058 (16.8%)* |
| Asthma | 1546 (24.6%) |
| Allergic rhinitis | 2073 (32.9%) |
| Main Location |  |
| *Face (including mouth lips and eyelids)* | *1495 (23.7%)* |
| *Hand* | *1331 (21.1%)* |
| *Leg* | *632 (10%)* |
| Generalized | 778 (12.4%) |
| Trunk | 655 (10.4%) |
| Anogenital | 449 (7.1%) |
| Flexural | 259 (4.1%) |
| Arm | 241 (3.8%) |
| Neck | 190 (3%) |

* Abbreviations: A, % patients with atopic dermatitis; A^(2)^, % patients age 40 and above; F, % patients with face dermatitis; H, % patients with hand dermatitis; L, % patients with leg dermatitis; M, % male patients; O, % patients with occupational dermatitis
